# Application of Least-Squares Support Vector Machines for Quantitative Evaluation of Known Contaminant in Water Distribution System Using Online Water Quality Parameters

**DOI:** 10.3390/s18040938

**Published:** 2018-03-22

**Authors:** Kexin Wang, Xiang Wen, Dibo Hou, Dezhan Tu, Naifu Zhu, Pingjie Huang, Guangxin Zhang, Hongjian Zhang

**Affiliations:** State Key Laboratory of Industrial Control Technology, College of Control Science and Engineering, Zhejiang University, Hangzhou 310027, China; 21532059@zju.edu.cn (K.W.); xwen@zju.edu.cn (X.W.); 21632031@zju.edu.cn (D.T.); znf@zju.edu.cn (N.Z.); huangpingjie@zju.edu.cn (P.H.); gxzhang@zju.edu.cn (G.Z.); hjzhang@iipc.zju.edu.cn (H.Z.)

**Keywords:** water quality early warning, quantitative evaluation, LS-SVM, conventional water-quality sensors

## Abstract

In water-quality, early warning systems and qualitative detection of contaminants are always challenging. There are a number of parameters that need to be measured which are not entirely linearly related to pollutant concentrations. Besides the complex correlations between variable water parameters that need to be analyzed also impairs the accuracy of quantitative detection. In aspects of these problems, the application of least-squares support vector machines (LS-SVM) is used to evaluate the water contamination and various conventional water quality sensors quantitatively. The various contaminations may cause different correlative responses of sensors, and also the degree of response is related to the concentration of the injected contaminant. Therefore to enhance the reliability and accuracy of water contamination detection a new method is proposed. In this method, a new relative response parameter is introduced to calculate the differences between water quality parameters and their baselines. A variety of regression models has been examined, as result of its high performance, the regression model based on genetic algorithm (GA) is combined with LS-SVM. In this paper, the practical application of the proposed method is considered, controlled experiments are designed, and data is collected from the experimental setup. The measured data is applied to analyze the water contamination concentration. The evaluation of results validated that the LS-SVM model can adapt to the local nonlinear variations between water quality parameters and contamination concentration with the excellent generalization ability and accuracy. The validity of the proposed approach in concentration evaluation for potassium ferricyanide is proven to be more than 0.5 mg/L in water distribution systems.

## 1. Introduction

With the progress of urbanization, there are increasing management problems concerning the pollution of water bodies or drinking water, which troubles many countries, especially developing ones. The idea of establishing an early warning system (EWS) to make the water supply system more robust against contamination events has been highlighted. In 2005, the United States Environmental Protection Agency (USEPA) introduced a framework for integrating early warning systems into water distribution systems to monitor, analyze, interpret and communicate data, which can protect public health [1]. Subsequently, numerous studies has been performed on water quality early warning technologies all over the world, including water quality sensor technologies, event detection algorithms, hydrological models, and decision-making systems (DSS) [2,3]. Establishing an early warning system has been recognized as an effective means of: (1) avoiding or reducing the impact of water contamination events; and (2) protecting water sources and ensuring the safety of drinking water [4].

In the past few years, a number of researchers have discussed the various contamination detection techniques. In EWS, the detection module plays a significant part to adopt online sensors to monitor water quality and detect contamination. The online conventional water quality sensor techniques of water events detection are mainly divided into three categories, artificial intelligence (AI), statistical approach, and data mining method [5] respectively. Relying on a fixed-length moving time window as well as a single water-quality parameter, the time series prediction makes statistical methods potentially inefficient in tracking the water quality data trends [6,7,8]. In terms of AI methods, they include support vector machines (SVM), regression trees, ensemble methods, Bayesian analysis and artificial neural networks (ANN), which are aimed at water quality data classification [5,7,9]. For example, Bucak and Kalik [10], and Bouamar and Ladjal [11] used SVM and ANN to classify water quality data into two classes: normal and anomalous. As for data mining, it is used to protect drinking water systems by combining various sensors measurement values and location information [6,8]. Moreover, for improving the detection of water-contamination events, data-fusion methods have been introduced. They can piece various types of information together, such as operational data [12], additional station-specific features [9], and data from multiple monitoring stations [13]. In 2005, Hall et al. [14] demonstrated that it is possible to detect changes in water-quality parameters by using real or near real-time sensors. Empirical evidence proves that conventional water quality indicators, including pH, conductivity, total nitrogen, free chlorine, and total organic carbon (TOC), are sensitive parameters of contaminants such as arsenic trioxide, nicotine, and *Escherichia coli*. Accordingly, the method of anomaly-based water-contamination event detection has gradually drawn the attention of many researchers. Besides conventional water quality indicators, various sensing technologies are used for vulnerability reliably of groundwater, river or water reservoir [15,16,17]. Among them, the biological stimulation technology, represented by the electronic tongue, has developed rapidly in recent years [18,19].

In addition to event detection and qualitative analysis, the quantitative evaluation of contaminant concentrations another critical part of water quality analysis and early warning, which plays an important role in contamination incident evaluation, pollution tracing, and source tracking. It involves a visual assessment of the degree of water pollution. Nevertheless, the existing quantitative evaluation method requires a lot of chemical analyses in a laboratory environment, which is time and resource consuming.

In recent years, quantitative characteristics of water parameters were widely investigated. Some studies have identified the quantitative characteristics of sensor responses by using hydrodynamic models and multifactorial data analysis technologies comprehensively. In a study by Yang et al. [20], a modified one-dimensional Danckwerts convection–dispersion–reaction model has numerically explained the observed chlorine residual loss for a “slug” of reactive contaminants which are instantaneously introduced into a drinking water pipe with wall demand that is assumed to be absent or negligible. The research results demonstrated that the change in water quality indicators occurs immediately after the injection of contaminant and becomes steady around the crest value. Meanwhile, it confirmed a linear relationship between water quality parameters and contaminants. In addition, by hydrodynamic models and time-frequency methods, Feng et al. [21] studied the relationship between contaminant concentration and variation of free chlorine. This model-based algorithm relies on a real-time hydraulic and water quality model to estimate water quality signature for comparing it with sensing water quality signals. Although, there are still gaps in the quantitative study of contaminant concentration online, the quantitative evaluation with conventional online sensors, compared with time-consuming and expensive off-line chemical analysis in the laboratory, is more suitable for sudden pollutant incident. 

Based on the least squares support vector machine (LS-SVM), a method for evaluating contaminant concentration quantitatively using different types of conventional water quality sensors for drinking water is described in this paper. The proposed method aims to quantitatively determine contaminant concentrations by exploring two attributes of responses from multiple sensors. One is the correlative relationship between responses from multiple sensors and different concentrations of the experimental contaminant; the other is the change value of water quality parameters relative to the baseline value caused by different contaminant concentrations. The proposed method is tested using online data from contaminant-dosing experiments in a laboratory. Thereafter, results of the method and problems caused by disturbance of water quality parameters and equipment are discussed. In this paper, the method is tested by online data from contaminant-dosing experiments in the laboratory. Afterwards, results of the method and problems triggered by equipment and water quality parameter disturbances are discussed.

## 2. Methodology

**Figure 1:**
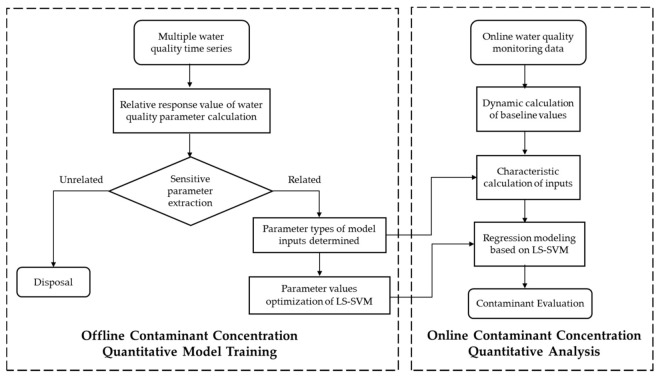
Schematic of a method for quantitative analysis of contaminant concentration using LS-SVM.

Figure 1 shows the algorithm flowchart in this paper. There are two steps: firstly trained by the data from online, the sensitive parameters are extracted off-line, then an on-line characteristic calculation is conducted to establish the LS-SVM regression model, consequently, draw the evaluation of contaminants.

### 2.1. Least-Squares Support Vector Machines (LS-SVM)

SVM and Support Vector Regression (SVR) have been successfully used in hydraulic engineering issues such as wastewater quality indicator prediction [22], water quality management [23], rainfall-runoff modeling in urban drainage [24], flood stage forecasting [25], monthly streamflow forecasting [26], and prediction of air entrainment rate as well as aeration efficiency of weirs [27]. Liu et al. [28] adopted SVR with GA optimization to predict aquaculture water quality, which to some extent weakened the impact brought by the nonlinearity and non-stationarity of water quality indicator series. The generalization performance between SVR and ANN was compared by Behzad et al. [29] to predict one-day lead stream flow, which proved SVM was more effective for water resources management. 

Vapnik [30] introduced a reliable solution for classifying and recognizing patterns. As an extension of SVM, LS-SVM tends to calculate the loss function with the linear least-squares criteria [31]. It is supposed that a set of data $\left\{x_{i},y_{i}\right\},i=1,2,\ldots ,n$ exist, with input data *x_i_* and corresponding target *y_i_*. In the LS-SVM model, the error *ξ_i_* quadratic norm presents the loss function of LS-SVM. Optimization problem can be reflected as below:

(1) $$\min\underset{w,b,\xi }{J}\left(w,\xi \right)=\frac{1}{2}w^{T}w+\gamma \frac{1}{2}\sum_{i=1}^{n}\xi_{i}{}^{2},$$

under the equality constraints:

(2) $$y_{i}=w^{T}\phi (x_{i})+b+\xi_{i}{}^{2},\;i=1,\ldots n.$$

the model of LSSVM is expressed as below:

(3) $$f(x)=w^{T}\phi (x)+b,$$

where $\frac{1}{2}w^{T}w$ means a flatness measurement function, *J* denotes the objective function, ξi=αiγ refers to the error variable; γ stands for a punishment influential factor of the tradeoff between the model flatness and the training error, and the nonlinear mapping ϕ depicts inputting data to a space with high-dimensional characteristics, in which a linear regression problem can be solved and obtained; in addition, b is the bias; *w* presents a weight vector of dimension similar to the feature space.

Thus, *L* is presented as:

(4) $$L(w,b,\xi ,\alpha )=\frac{1}{2}w^{T}w+\gamma \frac{1}{2}\sum_{i=1}^{n}\xi_{i}{}^{2}-\sum_{i=1}^{n}\alpha_{i}(\xi_{i}-y_{i}+w^{T}\phi (x_{i})+b),$$

where *α_i_* refers to the Lagrange multiplier, leading to the Karush–Kuhn–Tucker conditions for optimality as below:

(5) $$\left\{ \begin{array}{l} \frac{\partial L}{\partial w}=0\to w=\sum_{i=1}^{n}\alpha_{i}\phi (x_{i})\\ \frac{\partial L}{\partial b}=0\to \sum_{i=1}^{n}\alpha_{i}=0\\ \frac{\partial L}{\partial \xi_{i}}=0\to \alpha_{i}=\gamma \xi_{i}\\ \frac{\partial L}{\partial \alpha_{i}}=0\to w^{T}\phi (x_{i})+b+\xi_{i}-y_{i}=0 \end{array}\right.\;i=1,\ldots n.$$

When variables *w* and *ξ_i_* are eliminated, transforming the optimization problem into the linear solution as below:

(6) $$\left[\begin{array}{cc} 0 & Q^{T}\\ Q & K+C^{-1}I \end{array}\right]\left[\begin{array}{c} b\\ A \end{array}\right]=\left[\begin{array}{c} 0\\ Y \end{array}\right],$$

where $Q={\left[1,1,\ldots ,1\right]}^{T}$, $A={\left[\alpha_{1},\alpha_{2},\ldots ,\alpha_{m}\right]}^{T}$, and $Y={\left[y_{1},y_{2},\ldots ,y_{m}\right]}^{T}$.

Based on the Mercer’s condition, it is feasible to set the Kernel function as below:

(7) $$K(x_{i},x_{j})=\phi {\left(x_{i}\right)}^{T}\cdot \phi (x_{j}).$$

The resulting LSSVM model used in function estimation is:

(8) $$f(x)=\sum_{i=1}^{n}\alpha_{i}K(x_{j},x_{i})+b.$$

The Mercer kernel function *K*(*x_i_,y_i_*) has several different types, such as sigmoid, polynomial, and radial basis function (RBF). RBF is a common option for the kernel function because of an excellent overall performance and fewer parameters required [32]. Therefore, this study took RBF as the kernel function, which is expressed as follows:

(9) K(xi,xj)=exp{−‖xj−xi‖22σ2}.

Consequently, it is necessary to choose two parameters from the LS-SVM model: and the punishment factor *γ* and the bandwidth of the Gaussian RBF kernel *σ*. The bandwidth of the Gaussian RBF kernel controls the complexity of the final solution. The punishment factor is to adjust the equilibrium relationship between empirical risk and the regularization part, thus determining the penalty to the error square. The selection of very small *γ* or very large *σ* lead to underfitting of the regression machine. Conversely, overfitting may occur in the process of regression, decreasing the generalization of the regression.

### 2.2. Quantitative Evaluation of Known Contaminant by LSSVM

The application of LSSVM weakens the influence caused by the local nonlinearity of parameters as well as the disturbance of water environment, resulting in the realization of the quantitative evaluation. Descriptions of the method are as below.

#### 2.2.1. Procedure for Quantitative Evaluation of Known Contaminant

Different contaminants may cause different sensor responses, which can be used as inputs for a regression model. To establish the LS-SVM regression model, its inputs should be determined, including the parameter types of inputs and input values. Both are calculated by multiple water-quality parameters and related data in offline situations; thus, the LS-SVM regression model can achieve sensitive parameter extraction and establish an input matrix. In modeling, parameter optimization should be conducted by four different optimization methods to obtain the best regression performance. All of the previously mentioned details are part of the offline section to prepare for the online evaluation.

In the online part, based on the detection value of online monitoring instruments and the LS-SVM regression model for the known contaminant, the baseline value and characteristic value are calculated in real time. Then, the LS-SVM regression model is tested to evaluate the given contaminant concentration in drinking water, and different regression models with different input dimensions are compared. Figure 1 shows the algorithm flowchart of the given contaminant concentration evaluation based on LS-SVM. Each regression model for a designated pollutant in the regression model library is individually established.

#### 2.2.2. Sensitive Parameter Extraction

Firstly, the concept of relative response value to calculate the differences between water quality parameters and their baselines was introduced. Then, the change of the water quality parameters was taken as the result of contaminant introduction, which can be applied in analyzing the contaminant concentration quantitatively. Through the utilization of the maximum value of each sensor response data from one experiment, the relative response value of each parameter in the training set can be calculated by. Afterwards, the baseline value is subtracted before introducing the contaminant. 

However, incomplete time sequence values of water event make offline data processing infeasible in this application background. A traditional time-series forecasting method known as moving average can determine the baseline value of each water quality parameter before contaminant introduction. The window size value is set as 30, an empirical value. The length of the sliding window is set to ensure the time sequence in the partition window contains characteristic information as much as possible, and the data quantity can be controlled effectively to avoid high computational complexity.

The relative response values in each contaminant introduction are used to calculate the Pearson correlation coefficients for each sensor with potassium ferricyanide concentration. The Pearson correlation coefficient is used to measure the correlation between two variables X and Y, the value varies between −1 and 1. The closer the absolute values of the Pearson correlation coefficients of the two variables are to 1, the more linear they are with each other. If the Pearson correlation coefficient is small, the corresponding water quality parameter should be disposed of. According to the previous study [33], the threshold of such coefficient is chosen as 0.35 in this paper. The empirical value may affect the dimensions of the input, which will be discussed in this paper.

#### 2.2.3. Parameter Optimization

LS-SVM is believed to be very effective in forecasting regression. Especially it can determine the values of the punishment factor *γ* and the bandwidth of the Gaussian RBF kernel σ by applying proper metaheuristic algorithms. However, there is no unified method of optimizing the LSSVM parameters. The main idea is to set *γ* and *σ* in certain ranges, using leave-one-out cross validation [34] or K-fold cross validation [35] to obtain the accuracy of regression modeling with the training set and selected *γ* and *σ*. Finally, *γ* and *σ* are chosen as model parameters for achieving the best regression performance. In the simple LS-SVM algorithm, *γ* is 1 and σ=1l, where *l* represents the dimensionality of the input training data. 

LS-SVMLab v1.7 is a Matlab toolbox for LSSVM developed by De Brabanter et al. [36], which is used for optimizing LS-SVM algorithm in this study. In this version, the parameters are tuned in two phases. First, based on certain criteria, suitable parameters will be confirmed by a global state-of-the-art optimized technique, combined with motivated annealing (CSA) [37]. It has been proved that compared with multi-start gradient descent optimization, CSA is more efficient [38]. Due to the reduction in the sensitivity of the algorithm to the initialization parameters, efficiency improvement in optimization can be achieved, and maintain the quasi-optimal running of the optimization process [39]. By default, CSA applies five multiple starters [40]. Second, a second optimization procedure is used to these parameters for performing a fine tuning step. Optimization algorithms such as simplex, particle swarm optimization (PSO) grid search (GS), and genetic algorithm (GA) are introduced to obtain the best punishment factor and kernel bandwidth in the second optimization procedure. Thus, the LS-SVM regression modeling is conducted to compae the results.

The difference between the real output of the model and the expected output (concentration at sensors of solution mixed with drinking water and original configured contaminant solution in a laboratory) has been taken as the error, for measuring the predicting accuracy of LSSVM model, the outputs are represented in separate ways. The uncertainty of future predictions was estimated with the root-mean-square error of prediction (RMSEP) (Equation (10)) and squared correlation coefficient (r^2^) (Equation (11)):

(10) $$RMSEP=\sqrt{\frac{\sum_{i}{\left(y_{i}-\overset{\wedge }{y_{i}}\right)}^{2}}{I}},$$

(11) $$r^{2}=\frac{{\left(I\sum_{i}y_{i}\cdot \overset{\wedge }{y_{i}}-\sum_{i}y_{i}\cdot \sum_{i}\overset{\wedge }{y_{i}}\right)}^{2}}{\left(I\sum_{i}\overset{\wedge }{y_{i}{}^{2}}-{\left(\sum_{i}\overset{\wedge }{y_{i}}\right)}^{2}\right)\cdot \left(I\sum_{i}y_{i}^{2}-{\left(\sum_{i}y_{i}\right)}^{2}\right)},$$

In Equations (10) and (11), *I* stands for the number of testing samples, $\overset{\wedge }{y_{i}}$ refers to the predicted value of contaminant concentration estimated by the regression analysis method mentioned previously, and *y_i_* is the true concentration of the contaminant to which the conventional water-quality sensors respond.

## 3. Experiments Methodology

### 3.1. Experimental Design

**Figure 2:**
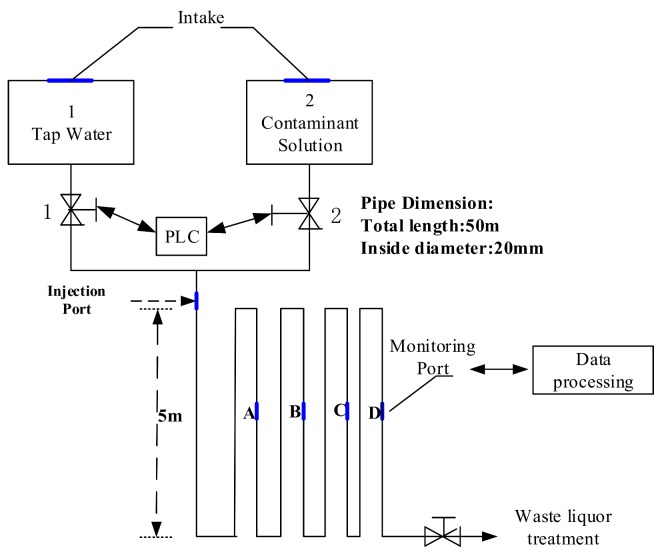
Experimental setup of a small-scale distribution pipe device. PLC: programmable logic controller. Water quality can be monitored at points A, B, C, and D.

**Figure 3:**
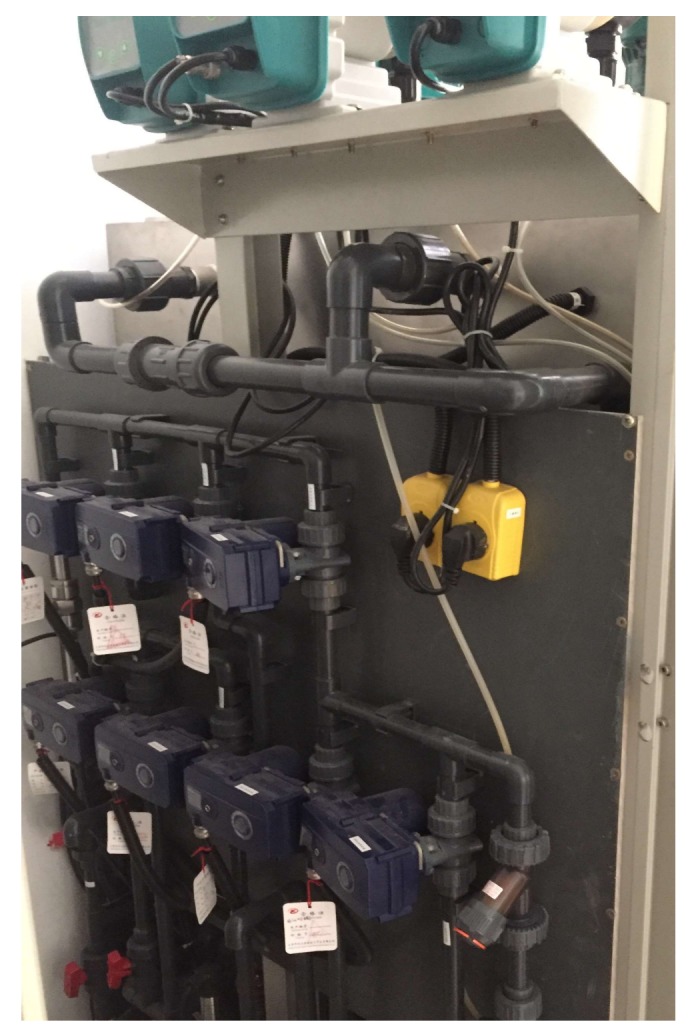
Pipeline distribution control system.

Contaminant injection in water, transport, monitoring, and detection testing was conducted in an experimental water-distribution system (Figure 2 and Figure 3). The water quality indicators are obtained in this experimental device including residual chlorine, total chlorine, chloride, oxidation-reduction potential (ORP), total organic carbon (TOC), PH, nitrate-nitrogen, ammonia nitrogen, turbidity, temperature, conductivity, chemical oxygen demand (COD) and dissolved oxygen (DO), etc. 

The experimental water system contains a ductile iron water-distribution pipe with a length of 50 m and an internal diameter of 20 mm. Besides, the system is composed of a programmable logic controller (PLC), contaminant monitoring points, contaminant injection point, solenoid valve, contaminant solution tank, and one tap-water tank. The PLC is to control the contaminant solution into the pipe and the flow rates of tap water. There are two contaminant injection methods: namely, injection through a contaminant injection point and using a contaminant solution tank for injection. The first method was applied to simulate a real pollution incident and obtain a training set because it provided a relatively stable intensity of an injection by setting the mixing proportion with tap water.

At the beginning of the whole experiment, tap water, whose flow can be controlled by valve, was run through the experimental pipe for at least 60 min to ensure that the system worked normally. The contaminant solutions of potassium ferricyanide were prepared with different concentrations separately. Before each injection, the experimental system had kept running for at least 25 min to establish a baseline. Thereafter, the contaminant solution with different concentrations was individually injected into the pipe of tap water at the contaminant injection point. Subsequently, the changes of water quality were measured from the injection port at downstream stations. The frequency of water-quality sampling at monitoring point D was measured every minute with the sensors listed in Table 1. 

The experiments are mostly composed of two parts: establishing the training set and obtaining the water quality data with pollution events. According to experimental test results before the training, upper and lower limits of contamination concentrations can be roughly acquired when all water quality sensors work effectively. The concentration gradient Δ of the training set is determined by the sample amount. The detection values of each water quality parameter can be recorded depending on the equal intervals of contaminant concentration. In addition, the detection values of drinking water are recorded as a baseline. Three experiments were conducted for each contamination sample with different specified concentrations to obtain the original water parameters of the training set. The even data will cover the normal data to form the original water parameters of the test set.

In addition, mimicking a pollution event in a distribution system is complicated because of difficulty in environmental monitoring and safety. Therefore, experiments and simulations are used to solve the problem. The experiment structure is composed of event data detection and normal data detection. Through a simulated water pollution event, contaminant concentration in water is obtained with a given time interval. Moreover, based on chemical experiments, the pollution condition is reproduced and the detection of relative water quality parameters is performed. Also, the detection is conducted in the distribution system with the same time interval. The former data are called event data and the latter are called normal data. Finally, the normal data are overlaid with the event data to form the original water parameters of the test set.

When operating in a single-pass contaminant mode, the contaminant solution is pumped by a peristaltic pump to a pipe that is connected to the tank and the sensor. According to the current tap water flow, mixed proportion of tap water, and contaminant solution set in the upper computer, PLC controls the injection flow of the contaminant solution. The mixing ratio is set at 0.5%, which allows the injection of 500 mL contaminant solution to be sustained for approximately 20 min.

In the regression process, the real concentration of contaminant solution, which flows through online water quality sensors and then directly into a designated sewage treatment pool, is the calculated value of the initial injected contaminant concentration mixed with tap water by the setting ratio. After the injection of contaminant solution, the pipeline still has running water, so that the sensor responses revert to the baseline level.

### 3.2. Contaminants Investigated

Specific quantities of different contaminants were injected into the system. According to supplements and contrasts from previous studies by Yang et al. [40] and Hall et al. [41], the correlative response of sensors for five inorganic compounds and one organic compound selected in the present study is listed in Table 2. 

Based on the survey of water pollution events in the water supply systems of Chinese urban area for the past two decades, the contaminants were confirmed. These contaminants consist of three kinds of pollutants that are the most common in agricultural use (urea), chemical industry use (sodium nitrite aldicarb, and potassium biphthalate), and heavy metals (cupric sulfate). The selection of them was consistent with the national standards of China concerning drinking water quality in GB3838-2002.

Table 2 summarizes the different responding sensors for injections of different contaminants. Other studies have revealed a similar phenomenon. In 2007, Hall et al. [40] reported the influence of nine different contaminants on conventional water quality parameters through analysis based on experiments; the report also proved that particular pollutants could be reflected by water-quality monitoring indicators. Szabo et al. [42] utilized a single-pass pipe to simulate a drinking-water distribution system for a study between contaminants and water quality parameters, in which similar results were obtained. In 2009, Yang et al. [40] conducted a sensor response experiment for 11 types of contaminants and observed more than one sensor responding to each tested contaminant.

As shown in Table 2, differently tested contaminations may cause different correlative responses of sensors. And the degree of the aforementioned response base value is employed to establish an LS-SVM regression model, which can be used to evaluate the same contaminant with an unknown concentration in water distribution system. The studies mentioned verified the assumption of the proposed method.

Potassium ferricyanide is used to explain the quantitative evaluation method presented in this study with online sensors. It is primarily used in manufacturing, paints, inks, pigments, pharmaceuticals and food additives. The main toxic effect on human is kidney damage. The sample concentration gradient depends on the sample amount and the detection range of the online water-quality sensor.

## 4. Results and Discussions 

### 4.1. Correlative Responses

**Figure 4:**
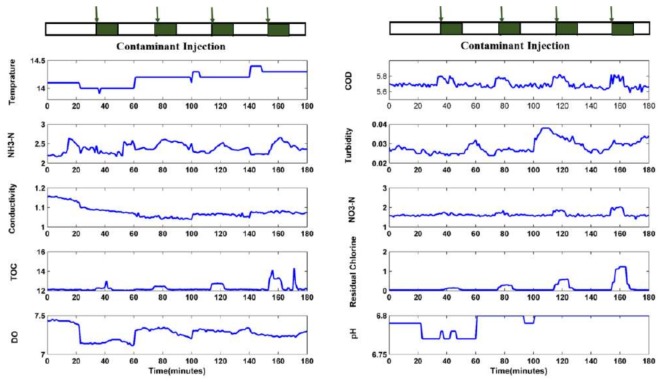
Sensor responses for potassium ferricyanide (concentrations: 1.0, 2.0, 4.0, and 8.0 mg L^−1^).

As an example, Figure 4 shows the experiment results associated with potassium ferricyanide. Throughout the experiment, potassium ferricyanide solutions with concentrations of 1.0 mg L^−1^, 2.0 mg L^−1^, 4.0 mg L^−1^, and 8.0 mg L^−1^ were tested in turn. The concentrations are at the sensors, and are not the initial injected contaminant concentration via the peristaltic pump. This condition is indicated by solid green bars at the top of Figure 4. In the figure, COD, NO_3_-N, TOC, and residual chlorine increase because of the presence of potassium ferricyanide. In addition, the response of residual chlorine is relatively slow but stable; however, random fluctuations still occur in the stable state of the other three indicators. When the concentration of potassium ferricyanide at the sensors is greater than 1.0 mg/L, the numerical value of NH_3_-N relative to the background also has an obvious upward trend. However, when the contaminant concentration is 1.0 mg/L or 0.5 mg/L or even less, the numerical value of the NH_3_-N is irregular, which shows the easily variable characteristic of water quality parameters. Sensor responses show correlative relationships, especially for COD, NO_3_-N, TOC, and residual chlorine, as well as reflect the response degree because of the induction of different contaminant concentrations. 

Obviously, the response amplitudes of sensors are related to the contaminant concentrations. This fact suggests that correlative response and response amplitude are caused by the introduction of different contaminant concentrations and implies that this type of phenomenon can be utilized for quantitative evaluation of contaminants. In order to justify the feasibility and applicability of the proposed method, offline experiments of potassium ferricyanide were conducted before online potassium ferricyanide injection experiments. Although types and detection methods of water quality sensors used in offline experiments are different from those of online ones, the correlative responses of pH, conductivity, residual chlorine, total chlorine, and NH3-N are also presented in the offline experiments of potassium ferricyanide. The responses of sensors are similar to the case of online experiments, which indicates that the proposed approach can also be utilized for quantitative evaluation in offline cases. By comparing with results from other types of contaminants, the response curves are clearly contaminant-specific. Obviously, obtaining parameters that will change with the contaminant concentration is necessary, while the others are disposed of. In other words, sensitive parameter extraction is needed to determine the input parameters of the regression model.

### 4.2. Sensitive Parameter Extraction

Because the detection upper bound of NO_3_-N is 2.0 mg/L, the maximum concentration of potassium ferricyanide in the training set is 18 mg/L. In Figure 4, with the change in contaminant concentration, the change values relative to the baseline values of the water quality sensors are different. The change values of water quality parameters can be considered as the result of contaminant introduction and can be used for the quantitative analysis of contaminant concentration. The concept of relative response value was introduced to calculate the differences between water quality parameters and their baselines. Ideally, the maximum response value should be stable and completely caused by contaminant introduction, and not the superposition caused by water quality noise. For example, after the introduction of 1 mg/L and 8 mg/L potassium ferricyanide, significant single-point mutation noises appear in the TOC response data (Figure 4). The maximum response value of NO_3_-N occurs at the end of the response time, while no ideal stationary change appears in the response values of COD and NH_3_-N. Table 3 presents the relative response values of eight water quality parameters caused by potassium ferricyanide introductions with different concentrations. Other data processing methods for baseline values of water quality sensors are discussed in a later part of this paper.

The Pearson correlation coefficients for each sensor with potassium ferricyanide concentrations are calculated (Table 3). In the table, the calculated Pearson correlation coefficients are 0.0746 (conductivity), 0.0886 (turbidity), 0.0533 (DO), 0.3748 (COD), 0.6080 (TOC), 0.7690 (NH_3_-N), 0.7541 (NO_3_-N), and 0.9997 (residual chlorine). In the regression model process of potassium ferricyanide, COD, TOC, NH_3_-N, NO_3_-N, and residual chlorine are chosen as the model inputs according to the description in Section 2.2.3.

**Figure 5:**
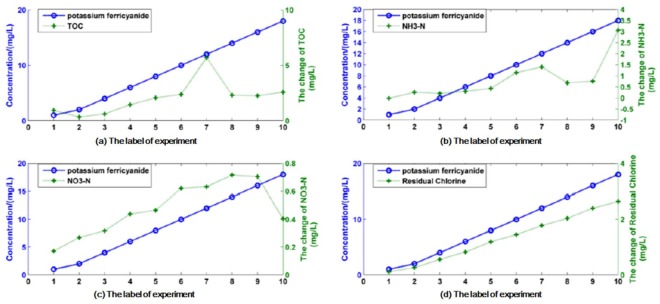
Relative response values of four parameters with concentration of potassium ferricyanide: (**a**) TOC; (**b**) NH_3_-N; (**c**) NO_3_-N; (**d**) residual chlorine change.

Figure 5 presents that the relative response values of TOC, NH_3_-N, NO_3_-N, and residual chlorine change with the concentration of potassium ferricyanide. The values of the abscissa represent the labels of experiments, and the left values of ordinate represent the concentrations values of potassium ferricyanide while the right values of ordinate represent the change values of TOC, NH_3_-N, NO_3_-N, and residual chlorine.

In the figure, a correlation exists between TOC and potassium ferricyanide. However, the Pearson correlation coefficient is relatively small because of the noise introduced by the seventh sample; and the relative response value of TOC in subsequent samples tend to saturate. The good linear correlation between residual chlorine and potassium ferricyanide concentration largely depends on the detection method for residual chlorine. Residual chlorine is measured once every 2.5 min but recorded every minute. It can be seen from the figure that the strength of the correlation between different water quality indicators and contaminant concentrations is different, and the reason for this difference is that there is a local nonlinearity in the relative response.

### 4.3. Modeling and Test

A simple method for model selection is to randomly divide the data set into three parts, namely training set, validation set, and test set. The training set is used to train the model, the validation set is used to select the model, and the test set is used to ultimately evaluate the learning method. In the different complexity models learned, the model with the smallest prediction error for the verification set is selected. Since there are enough data in the validation set, it is also effective to use it to select models. However, in practice, data is often insufficient. In order to select a good model, cross-validation can be used. A simple cross-validation method is used here. Firstly divide the given data into two parts randomly, one part as a training set and the other as a test set. Then use the training set to train the model under different conditions to obtain different models. The test error of each model is evaluated on the test set, and the model with the smallest test error is selected.

After the training set is established, the test set needs to be determined. To improve the simulation of the diffusion of a contaminant in the distribution system, the simulation of contaminant release is performed. Based on the simulation results, chemical experiments are conducted. Table 4 presents the real concentrations of each sample and the relative response values of model input parameters in the test set.

According to the modeling process mentioned, the complete training set data are applied for the tuning of *σ* and *γ* first by using LS-SVMLab v1.7. Then, the LS-SVM regression model with RBF is conducted by using the obtained model parameters and the training set data. Thereafter, the regression model is tested using the test data. In the present study, the initial parameters of the GA-LSSVM and PSO-LSSVM are given as follows: the maximum iteration number *maxgen* = 200, the population size *sizepop* = 20, the range of *σ* ⊂ [0, 10 × 104], the range of *γ* ⊂ [0, 10 × 103], and the object accuracy *mse* (mean square error) = 0.01. The step values of *σ* and *γ* in GS-LSSVM are both 0.8. The initial parameters adopted in simplex are taken by default in the Matlab toolbox. Simplex, GS, GA, and PSO are introduced in the second optimization procedure to obtain the best *σ* and *γ*.

**Figure 6:**
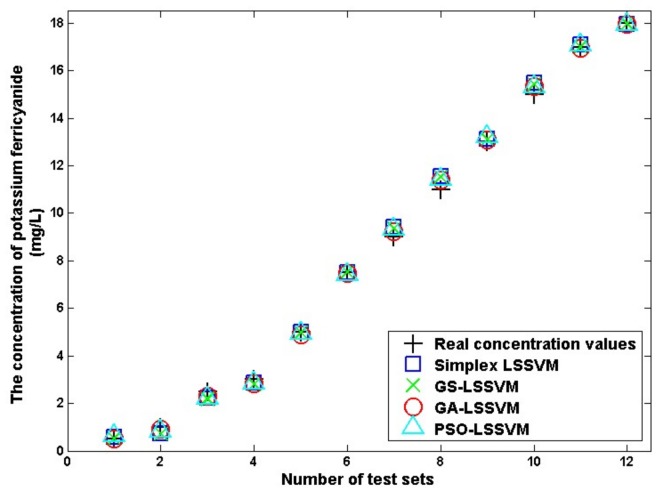
Illustration of comparison between real value and prediction value based on four parameter optimization algorithms.

Figure 6 shows an illustration of a visual comparison between the real value and prediction value by LS-SVM regression models with four parameter optimization algorithms. The values of the abscissa represent the labels of testing set, and the values of ordinate represent the concentrations values (both real concentration values and prediction values based on different parameter optimization algorithms) of contaminants.

The curve marked with plus signs is the real concentration value of potassium ferricyanide, while the curves marked with squares, crosses, and triangles are the prediction values by the simplex-LSSVM, GS-LSSVM, and PSO-LS-SVM models, respectively. The result predicted by the GA-LSSVM model is marked with circles.

Table 5 presents the prediction performance of the test dataset in Table 4 for each LS-SVM model. The RMSEP and r^2^ of the testing set were obtained (Table 5) using Equations (3) and (4).

In the table, RMSEP and r^2^ of the regression result for the testing dataset are acceptable when parameter optimization methods were used to obtain the best parameters for the LS-SVM training, whereas the model obtained by GA-LSSVM has the best generalization ability because of the high parallelism degree and strong adaptability of the genetic algorithm.

To test the performance of the described approach, two other models, LSSVM and multiple linear regression models, are also developed for comparison purposes. The LS-SVM model obtained by using the parameter optimization method performed better in terms of accuracy and generalization ability. In detail, the LS-SVM model produces a RMSEP of 1.3158 and an r^2^ of 0.9712, while the multiple linear regression models generate a RMSEP of 2.5327 and an r^2^ of 0.8531. As the RMSE indicator illustrates, compared with that of LS-SVM with default model parameters, the predictive error of PSO-LSSVM and GA-LSSVM decreases by 82.4% and 85.9%, respectively, proving that the proposed method provides good concentration prediction accuracy for quantitative evaluation of a known contaminant.

The aforementioned regression models were built with a five-dimension vector input. During the experiment, NH_3_-N had minimal response to the contaminant solution, of which the concentration was 1 mg/L, 0.5 mg/L, or even less. The relative response value recorded in Table 4 was 0.9365 mg/L when the concentration of potassium ferricyanide was 0.5 mg/L, which was possibly an illusion caused by noise fluctuation of water quality after response time. These potential errors were not eliminated because noise may arise anytime in a real-time sense, and the model obtained by the proposed method should have the robustness to handle small errors in one dimension of a five-dimension input model.

### 4.4. Analysis of Noise Source and Detection Limit

A common question for the regression modeling problem is how to discriminate the influence of environmental noise included in characteristics and how to determine model input dimensions.

**Figure 7:**
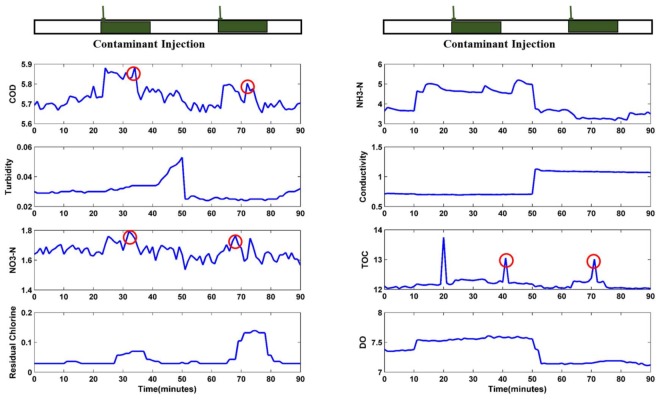
Sensor responses for potassium ferricyanide (concentrations: 0.5 and 1.0 mg L^−1^).

Figure 7 shows the response curve for potassium ferricyanide with concentrations of 0.5 and 1.0 mg L^−1^. Introduced by equipment noises mainly, lots of peaks and troughs exist in the graphs of turbidity, conductivity, and DO. These noises are independent and not related to contamination injections. This result is verified by the weak Pearson correlation coefficients for turbidity, conductivity, and DO (Table 3), and also indicates that turbidity, conductivity, and DO does not respond to the presence of potassium ferricyanide.

As the input of the LS-SVM regression model, the relative response value is also a characteristic value extracted from the experimental data. The extraction refers to two parameters: baseline value and maximum response value, both of which may be responsible for deviation of results. For example, as shown in Figure 7 some peaks and troughs during response time (e.g., marked in the graph of COD, TOC, and NO_3_-N) shifted significantly from the previous reading because of equipment noise. This type of shift is difficult to predict, and a significant deviation between response value and baseline value occurs for real-time quantitative analysis. The baseline value relates to the window size and right boundary of the moving average model. Quantitative evaluation of water quality detection occurs immediately after the qualitative analysis, which includes contamination detection and contamination identification. When the detection decision is made, the right boundary of the moving average model is determined.

In the present study, we focus on the quantitative evaluation of contaminant concentrations but not contamination event detection; thus, the injection time of contaminations is chosen as the right boundary. Meanwhile, window size denotes the number of data involved in the calculation of the baseline value. Given the relative stability of the baseline, the window size need not be extremely large. With the autoregressive moving average method reported by Hou [43] for comparison, a greater demand for historical data is observed because the input data of the autoregressive moving model should be thousands of orders of magnitude.

As mentioned in the last section, NH_3_-N had a minimal response to the contaminant solution when the concentration was 1 mg/L, 0.5 mg/L, or even less. As predicted for the two concentrations in the test set, only four of the five dimension inputs were useful, while NH_3_-N may change to an abnormal input, which caused prediction errors. Meanwhile, 0.5 mg/L and 1 mg/L were out of the range of the training set, which was from 2 mg/L to 18 mg/L, leading to several regression errors. Because of the adaptive and autocorrelation analysis of GA, GA-LSSVM has better generalization ability. However, regardless of what optimization method is used, a lower detection limit is defined, which is 0.5 mg/L in our study. Improving the generalization ability to improve the prediction accuracy around the detection limit by sacrificing the accuracy of the model is unnecessary.

### 4.5. Effects of Input Dimensions

A reasonable construction of the input vector is the premise of applying LSSVM to predict the time series, and the influence of different construction methods on prediction precision and efficiency is affected significantly. In practical application, proposing a generally applicable method of constructing the input vector for all types of time series with different characteristics is difficult. In the present study, every dimension of the model input has a different calculated Pearson correlation coefficient with potassium ferricyanide concentration. COD, with a Pearson correlation coefficient of 0.3748, was also chosen as an input. Input dimensions might influence the performance of the quantitative evaluation. To understand this, given a specified dimension value, parameters with greater Pearson correlation coefficient are chosen as model input for model reconstruction and retesting. 

Table 6 presents the evaluation performance of the GA-LSSVM model built with different input dimensions. It demonstrates that the proposed method can be effectively used for quantitative analysis regardless of what the dimensions are. It also implies that the GA-LSSVM model with one or two dimensions has better performance for the given test set in this study. Residual chlorine is chosen as the only input of the GA-LSSVM model with one dimension because of its significant Pearson correlation coefficient. However, the robustness of the GA-LSSVM model with one or two dimensions is weak because of has been recognized the reliance on the linear relationship between contaminant concentration and residual chlorine or NO_3_-N.

### 4.6. Analysis of Algorithm Reproducibility

**Figure 8:**
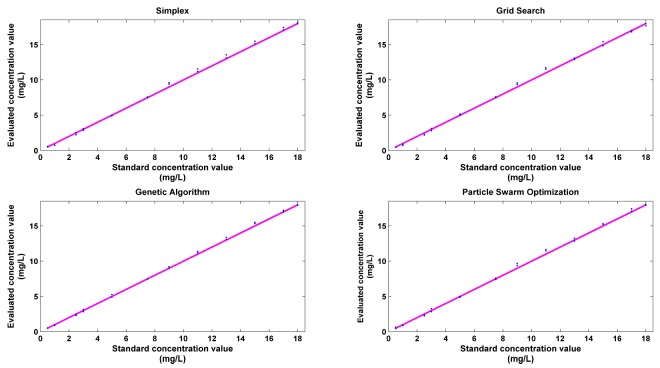
Correlation between standard concentration value and evaluated value modeled by LS-SVM.

Data from two sets of independent injection experiments were used to assess the reproducibility of the proposed quantitative evaluation method. The data will form a testing set to test the regression model previously achieved by using four different parameter optimization methods. The experimental conditions for the two groups of experiments are the same. In Figure 8, the concentrations of all 24 testing samples in the two groups are evaluated by four regression models. 

## 5. Conclusions

From the above analysis, this paper introduces the water-contamination quantitative evaluation method using multiple conventional water quality parameters, which comes to the following conclusions: 

Firstly, based on the phenomenon that different contaminations may cause different correlative responses of sensors and the degree of responses is related to the injected concentration of contamination, the experiment results imply that this type of phenomenon can be used for quantitative analysis of a known contamination incident in a water distribution system. The concept of relative response value is introduced to calculate the differences between water quality parameters and their baselines. 

Secondly, by utilizing LS-SVM, the presented method can reduce the effect of the limited number of training samples, adapt well to local nonlinear response of water quality parameters, and improve the prediction accuracy of pollutant samples at low concentrations.

Thirdly, according to the correlation coefficient calculated in the training set, the inputs of the LS-SVM regression model are chosen. In modeling, four different parameter optimization methods are used to optimize the penalty factor *γ* and the kernel width *σ*. The results illustrate that the regression model obtained by GA, which works well on adaptive capacity and auto-correlation analysis, shows the most effective performance in parameter optimization. 

Lastly, our findings indicate that the proposed quantitative evaluation method can effectively predict contamination concentration not less than 0.5 mg/L and use real-time monitoring of water quality. It is necessary to develop a credible library of contaminant-sensitive parameters for implementing the proposed quantitative evaluation method with respect to the drinking water systems coupled with EWS operation in real time.

## Figures and Tables

**Table 1:** Characterization of drinking water in current study.

Parameter	Concentration	Parameter	Concentration
Temperature	14.0 °C	pH	6.77
DO	6.950 mg L^−1^	Turbidity	0.026 NTU
COD	5.763 mg L^−1^	TOC	12.512 mg L^−1^
Conductivity	0.914 μs/cm	NH_3_-N	2.890 mg L^−1^
NO_3_-N	1.624 mg L^−1^	Residual chlorine	0.029 mg L^−1^

**Table 2:** The responding sensors to various contaminants.

Contaminant	Responding Sensors		
	Hall et al. [41]	Yang et al. [40]	Current Study
	Sensor Array	Sensor Array	Sensor Array
	A, B, D, E, G, H, I	A, B, C, D, F	A, E, F, G, H, I, J, K, L, M
	Drinking Water	Drinking Water	Drinking Water
Aldicarb	A, B, D, E, I	A, B, C, D	
Arsenic trioxide	A, B, D, G, H, I		
Colchicine		A, B, C, D	
Dicamba		D, F	
*E. coli*	A, B, E, H, I	A, B, C, D, F	
Glyphosate	A, C, D, E	A, B, C, D, F	
Malathion	A, D, E, I		
Mercuric chloride		A, C, D, F	
Nicotine	A, B, D, E, G, H	A, B, D, F	
Nutrient broth		A, B, C, D	
Terrific broth	A, B, D, E, I	A, B, D, F	
Typtic soy broth		A, B, C, D	
Ammonium citrate			H, K
Cupric sulfate			A, E, L
Potassium biphthalate			E, G, H, L
Potassium ferricyanide		B, C, D	A, E, G, H, L
Sodium nitrite			E, H, L
Urea			E, H

Note: A—residual chlorine; B—total chlorine; C—chloride; D—ORP; E—TOC; F—pH; G—nitrate-nitrogen; H—ammonia-nitrogen; I—turbidity; J—temperature; K—conductivity; L—COD; M—DO.

**Table 3:** Relative responding values and Pearson correlation coefficients (potassium ferricyanide, the 1st experiment).

Concentration	Conductivity	Turbidity	DO	COD	TOC	NH_3_-N	NO_3_-N	Residual Chlorine
(mg/L)	(us/cm)	(NTU)	(mg/L)	(mg/L)	(mg/L)	(mg/L)	(mg/L)	(mg/L)
1	−0.0305	0.00605	0.1145	0.11745	0.9335	−0.01	0.17185	0.1079
2	0.0009	0.00145	0.0415	0.12455	0.34565	0.2585	0.2684	0.261
4	0.0054	0.0035	0.0435	0.15275	0.6108	0.206	0.3177	0.557
6	−0.0058	0.0453	1.8745	0.2379	1.4498	0.3015	0.43915	0.824
8	0.0042	0.00425	0.064	0.176	2.06445	0.434	0.4661	1.1901
10	−0.04935	0.0581	1.1555	0.2599	2.36995	1.144	0.6202	1.44925
12	−0.06145	0.04615	2.329	0.6237	5.697	1.4025	0.631	1.77785
14	−0.0201	0.00005	−0.033	0.1799	2.31015	0.6875	0.71585	2.0392
16	0.01085	0.00585	−0.039	0.1869	2.25225	0.758	0.7045	2.40075
18	0.0139	0.00915	0.0755	0.25265	2.5757	3.0615	0.40465	2.64495
r_xy_	0.0746	0.0886	0.0533	0.3748	0.6080	0.7690	0.7541	0.9997

Note: r_xy-_Pearson correlation coefficients.

**Table 4:** Real concentrations and relative responding values in the test set.

Concentration	COD	TOC	NH_3_-N	NO_3_-N	Residual Chlorine
(mg/L)	(mg/L)	(mg/L)	(mg/L)	(mg/L)	(mg/L)
0.5	0.1722	0.8618	0.9365	0.1248	0.0394
1.0	0.11745	0.9335	−0.01	0.17185	0.1079
2.5	0.1264	0.4334	0.0015	0.19255	0.3125
3.0	0.1512	0.6176	0.4205	0.21635	0.3927
5.0	0.14375	0.87205	0.6935	0.3427	0.7004
7.5	0.1759	1.15385	0.3905	0.4474	1.0798
9.0	0.15305	1.43175	1.467	0.5052	1.3417
11.0	0.1947	1.5408	1.3285	0.5435	1.6668
13.0	0.3848	4.8059	2.905	0.6767	1.904
15.0	0.23175	2.07795	1.3135	0.7274	2.2522
17.0	0.28945	3.7201	2.068	0.656	2.5344
18.0	0.25265	2.5757	3.0615	0.40465	2.64495

**Table 5:** Prediction performance on the test dataset in Table 4.

True Value (mg/L)	Relative Error			
	Simplex	GS	GA	PSO
0.5	15.53%	0.24%	1.95%	27.96%
1.0	26.00%	28.57%	9.17%	13.97%
2.5	10.67%	12.64%	7.86%	10.41%
3.0	4.12%	6.12%	5.33%	4.52%
5.0	0.20%	0.63%	1.97%	0.51%
7.5	0.08%	0.06%	0.36%	1.12%
9.0	4.63%	4.12%	2.36%	3.95%
11.0	5.15%	4.73%	3.59%	4.09%
13.0	0.86%	0.73%	0.68%	1.95%
15.0	3.27%	2.89%	2.27%	2.15%
17.0	0.46%	0.04%	0.26%	0.53%
18.0	0.21%	0.18%	0.26%	0.27%
RMSEP	0.2762	0.2619	0.1855	0.2310
r^2^	0.9986	0.9988	0.9992	0.9990

**Table 6:** Impacts of input dimensions of GA-LSSVM on evaluation performance.

True Value	Relative Error				
	One Dimension	Two Dimensions	Three Dimensions	Four Dimensions	Five Dimensions
0.5	1.01%	1.66%	2.71%	2.97%	1.95%
1.0	2.16%	10.25%	9.18%	10.09%	9.17%
2.5	6.56%	6.43%	7.99%	7.41%	7.86%
3.0	4.52%	5.21%	6.55%	4.52%	5.33%
5.0	3.51%	1.95%	3.32%	1.95%	1.97%
7.5	0.16%	0.36%	0.89%	1.02%	0.36%
9.0	1.53%	2.32%	2.71%	3.65%	2.36%
11.0	3.98%	3.65%	3.67%	3.61%	3.59%
13.0	0.73%	0.72%	0.72%	0.77%	0.68%
15.0	2.08%	2.01%	2.62%	2.75%	2.27%
17.0	0.21%	0.23%	0.67%	0.54%	0.26%
18.0	0.25%	0.25%	0.53%	0.73%	0.26%
RMSEP	0.1793	0.1841	0.1902	0.1913	0.1855
r^2^	0.9994	0.9992	0.9991	0.9990	0.9992
